# 4420 Characterizing medical comorbidity prior to autism diagnosis in children before age two.

**DOI:** 10.1017/cts.2020.170

**Published:** 2020-07-29

**Authors:** Michelle D Failla, Kyle Schwartz, Shikha Chaganti, Tiffany G Woynaroski, Laurie Cutting, Bennett Landman, Carissa Cascio

**Affiliations:** 1 Vanderbilt University Medical Center; 2 Vanderbilt University

## Abstract

OBJECTIVES/GOALS: Autism spectrum disorder (ASD) is a developmental disorder with a high financial and personal burden. Individuals with ASD experience significant comorbid medical conditions. We identified conditions that appear prior to ASD diagnosis in order to potentially improve early screening practices. METHODS/STUDY POPULATION: We used electronic health record data from an anonymized database at Vanderbilt University Medical Center for individuals with ASD and matched controls to analyze comorbid conditions prior to an ASD diagnosis. Data were censored to include only individuals who first appeared in the databank prior to two years of age (n_total_ = 1551, n_controls_ = 976, n_ASD_ = 575). Comorbidities (~1800 conditions) were compared between the ASD and matched control group using a novel tool (pyPheWAS) to examine presence, count, and duration of comorbidities that occurred between 0-2 years old and before ASD diagnosis. RESULTS/ANTICIPATED RESULTS: Convulsions (p = 0.000404, ß = 0.807), constipation (p = 0.000789, ß = 0.894), and strabismus (p = 0.00243, ß = 1.155) were the most significant comorbid conditions prior to age 2 in individuals who would later be diagnosed with ASD. The group with ASD also had more visits associated with convulsions (p = 0.00511, ß = 0.195), diseases of the esophagus (p = 0.0117, ß = 1.675), and allergic reactions to food (p = 0.0119, ß = 0.540) prior to their diagnosis. The ASD group was also seen for a longer duration regarding convulsions (p = 0.000273, ß = 0.695), constipation (p = 0.00157, ß = 0.712), and malaise and fatigue (p = 0.00188, ß = 0.903) before ASD diagnosis. DISCUSSION/SIGNIFICANCE OF IMPACT: Precise comorbid condition profiles in early childhood may help uncover biomarkers leading to better prediction of a future ASD diagnosis. Medical conditions that precede the onset of measurable behavioral symptoms may enhance early screening, treatment, and intervention in ASD.

